# Food Safety Knowledge, Attitudes, and Practices Among Jordan Universities Students During the COVID-19 Pandemic

**DOI:** 10.3389/fpubh.2021.729816

**Published:** 2021-08-30

**Authors:** Tareq M. Osaili, Anas A. Al-Nabulsi, Asma' O. Taybeh

**Affiliations:** Department of Nutrition and Food Technology, Faculty of Agriculture, Jordan University of Science and Technology, Irbid, Jordan

**Keywords:** knowledge, attitude and practice, coronavirus, cross-contamination, sanitation, cooking, personal hygiene

## Abstract

**Objective:** This study aimed to evaluate food safety knowledge, attitudes and practices (KAP) amongst university students in Jordan and changes in food-related behaviors during the COVID-19 pandemic. Correlation between food safety KAP scores and general characteristics of university students was also evaluated.

**Methods:** A cross-sectional study was conducted where an Internet-based questionnaire was distributed through social media platforms. The sample consisted of 1,739 respondents from 29 Jordanian universities. The participants completed a 58-item questionnaire covering demographical characteristics and different food safety aspects which were namely “COVID-19 food-related attributes,” “food cooking and storage,” “personal hygiene.” “cross-contamination prevention/disinfection procedures.” and “restaurant hygiene.” Descriptive statistics, Chi square tests and binary logistic analysis were used to assess the data.

**Results:** The sample consisted of 67.2% females with a mean age of 21.3 ± 1.8 years. The average overall score of the tested aspects was 14.1/34.0 which corresponds to 41.3% of the questions being answered correctly. The percentage of correct answers of “COVID-19 food-related attributes,” “food cooking and storage,” “cross-contamination prevention/disinfection procedures,” “personal hygiene” and “restaurant hygiene” was 56.8, 36.6, 28.4, 44.6. and 36.9%, respectively. A significant (*P* <0.05) association between respondents' food safety KAP scores and gender, marital status, university degree, employment status, self-rating of food safety knowledge, and the source of food safety information.

**Conclusion:** University students in Jordan had insufficient KAP scores which is a concerning trend during the pandemic. Teaching fundamentals of food safety in the form of short courses/ lectures is recommended.

## Introduction

Outbreaks associated with food pose a great threat to public health. As per the World Health Organization (WHO), on an annual basis, about 600 million cases and 420,000 deaths are associated with the consumption of contaminated food and water (1). Foodborne outbreaks affect 48 million Americans, 4 million Canadians and 2.4 million Britons each year (2–4). A recent food poisoning outbreak in Jordan (Ain Al-Basha region) associated with contaminated Shawerma resulted in 700 infections and two deaths. The Shawerma was reported to be infected with *Enterococcus Faecalis* and *Campylobacter* (5).

Food can get contaminated during various stages of production, distribution, and storage (1). Measures commonly recommended to combat foodborne outbreaks include frequent/ correct technique of hand washing, appropriate cleansing of kitchen surfaces, storing food at suitable temperatures and the separation of raw and cooked food (6, 7).

The advent of COVID-19 has been reported to impact people's food preparation/ eating habits, consumer food safety awareness, food and hygiene related attitude and food purchasing behavior (8–11). The primary mode of transmission of the virus has been reported to be through person to person contact and via respiratory droplets generated by coughing or sneezing. Untrue to common belief, the COVID-19 virus is not foodborne (12). However, the entire affair revolving around food could act as a vehicle for transfer, for example, an infected individual could transfer the virus on to the food package, the utensils, table tops, cash, machinery or even via a simple handshake (13).

A previous study has reported that young adults (18 to 29 years old) are more likely to take the concept of food safety lightly (14). This could be because (with a probable exception of personnel whose predominant occupation revolves around food), this section of the population usually do not possess appropriate training/certifications (14). An area of concern is that it is this section of the population who tend to work in food service establishments (part- or full-time job) during their course of study. Moreover, they tend to cook for themselves and their colleagues (roommates/ friends etc.). They are also more likely to attend parties and take the seriousness of the pandemic lightly because of their belief of higher immunity in young adults (15). Hence, it is highly possible that they act as a vehicle for the transfer of this virus.

Multiple studies pertaining to food safety knowledge amongst different population strata have been conducted previously in Jordan (16–19). However, none of them have assessed the impact of the COVID-19 pandemic in university student's food safety knowledge, attitudes, and practices (KAP). Therefore, the present study aimed to (i) evaluate food safety KAP among Jordan universities students during the COVID-19 pandemic, (ii) determine the changes in food-related behaviors during the COVID-19 pandemic, and (iii) assess the correlation between food safety KAP scores and general characteristics of university students.

## Materials and Methods

### Study Design

A cross-sectional study was performed from March 2021 to April 2021 to assess food safety knowledge, attitudes, and practices amongst Jordan universities students during the COVID-19 pandemic. Any student currently studying at a Jordanian University (*n* = 29) above 18 years was considered to be eligible to take part in the study regardless of gender, academic year, full time/ part time or academic program. The total number of students in all public and private universities (inclusive of all degrees) at the beginning of the academic year of 2020–2021 was announced to be 322,349. The universities are spread throughout the country thereby increasing the representativeness of the sample.

### Questionnaire

The questionnaire was designed by adapting some existing questions from validated and reliable questionnaires used in prior studies pertaining to food safety (10, 16, 20–28). All authors went through the questionnaire in-tandem to discuss the questions that need to be included in the study. The questions were revised to remove the ambiguity and ensure that they were short and clear. This was done to avoid self-reported bias such as social desirability and acquiescent responding (29). The questionnaire was translated from English to Arabic. It was tested by four bilingual academicians specialized in food safety, for its understandability. The final questionnaire consisted of 58 items (Supplementary Material) starting with a cover page which explained the nature and purpose of the study besides the confidentiality statement. The Cronbach alpha coefficient value (used to check questionnaire reliability) was observed to be 0.774. The questionnaire was composed of four sections; demographic information (13 items), food safety knowledge (12 items), attitudes (7 items) and practices (26 items) during COVID-19. A combination of multiple-choice, true-false-not sure, and Likert-scale questions were used in the questionnaire. The questionnaire covered the following food safety aspects: “COVID-19 food-related attributes,” “food cooking and storage,” “personal hygiene,” “cross-contamination prevention/disinfection procedures,” and “restaurant hygiene.” The total score of students' knowledge, attitudes and practices was calculated by the summation of correct answers from each aspect. Each correct answer was given 1 point while incorrect and not sure answers were given a score of 0. Finally, the practice part consisted of questions pertaining to behavioral changes during the COVID-19 pandemic where the answer choices were “Less than before,” “About the same” and “More than before”, respectively. The final questionnaire draft was then piloted amongst students (*n* = 30). This involved completing the survey using different computers or phones at different locations. No further adjustments on the questions were needed as per the feedback.

### Data Collection

The data were collected via an Internet-based link (Google Forms). The invitation link was primarily distributed via students' groups on social media platforms namely Facebook and Twitter. The link was shared by the researchers, as well as willing participants—who forwarded it to other potential participants from the same or other universities (snowball approach).

On the first page of the questionnaire, participants had been informed that their participation was purely on a voluntary basis and their consent was taken prior to starting the questionnaire. The participants were given all information deemed necessary about the study on the consent form. They were informed of their right to withdraw from the survey at any time. There was no possibility of placing any undue pressure on the respondents as the survey had to be completed via an online link. All responses were kept confidential. The study and the protocol were approved by the Department of Nutrition and Food Technology (#26/2021) and Deanship of Graduate Studies (#7/2021) at Jordan University of Science and Technology.

### Data Analysis

All survey responses were exported from the Google Forms platform into SPSS Version 26.0 (SPSS Inc., USA) for analysis. Descriptive statistics of means, standard deviation, variation ratio, frequencies and percentages were used for variables as appropriate. Chi-square test was conducted to explore the difference between categorical variables. Binary logistic analysis was used to assess the contributing factors affecting students' knowledge, attitudes and practices (KAP) scores. A *p*-value < 0.05 was considered to be significant. A cut-off point of 50% was used to calculate the total participant score and a sufficient KAP score was considered when the participant correctly answered more than 50% of the questions.). A score of <50% was considered as inefficient knowledge, attitude and practice.

## Results

### Demographic Characteristics

A total of 1,739 students from 29 private and public universities in Jordan participated in this study. The sample consisted of 67.2% females with a mean age of 21.3 ± 1.8 years (Table 1). More than half (57.8%) of the participants studied at public universities. Most of the participants lived with their family (89.1%), did not work (77.4%), and helped in preparing food (83.6%). Only 12.0% of the participants rated themselves to have “excellent” knowledge of food safety. The main sources of food safety information were reported to be the internet (43.2%) (Table 1).

**Table 1:** Demographic characteristics of university students (*n* = 1739).

**Character**	**Frequency (%)**
Age [Mean, (range)]	21.3 (18–25)
**Gender**	
Female	1168 (67.2%)
Male	571 (32.8%)
**Marital status**	
Single	1630 (93.7%)
Married	109 (6.3%)
**University type**	
Public university	1006 (57.8%)
Private university	733 (42.2%)
**College**	
Humanities	559 (32.1%)
Scientific^*^	675 (38.8%)
Health^**^	505 (29.0%)
**University degree**	
Bachelors	1604 (92.2%)
Masters	131 (7.5%)
Doctorate	4 (0.2%)
**Year of study**	
First year	339 (19.5%)
Second year	297 (17.1%)
Third year	392 (22.5%)
Fourth year	513 (29.5%)
Fifth year	139 (8.0%)
Sixth year	59 (3.4%)
**Living with**	
Family	1550 (89.1%)
Roommate	80 (4.6%)
Alone	109 (6.3%)
**Employment**	
Do not work	1346 (77.4%)
Full time work	182 (10.5%)
Part time work	211 (12.1%)
**Monthly expenses**	
<100 JD (1 JD = 1.41$)	845 (48.6%)
100–300 JD	703 (40.4%)
>300 JD	191 (11.0%)
**Self-rating of food safety knowledge**	
Excellent	208 (12.0%)
Very good	625 (35.9%)
Good	768 (44.2%)
Weak	122 (7.0%)
Very weak	16 (0.9%)
**Source of food safety information**	
Courses/workshops	289 (16.6%)
Family	388 (22.3%)
Friends	26 (1.5%)
Healthcare professional	85 (4.9%)
Social media	167 (9.6%)
Internet	752 (43.2%)
Others^***^	32 (1.8%)
**Preparing/helping in preparing food**	
Yes	1453 (83.6%)
No	286 (16.4%)

* *Scientific college include: engineering, biological sciences, IT and agriculture.*

** *Health college include: medicine, dentistry, pharmacy and nursing.*

*** *Others include: dietitian, private sport trainer, self-information and experience*.

### Overall Food Safety Knowledge, Attitudes, and Practices Score of University Students During the COVID-19 Pandemic

**Figure 1:**
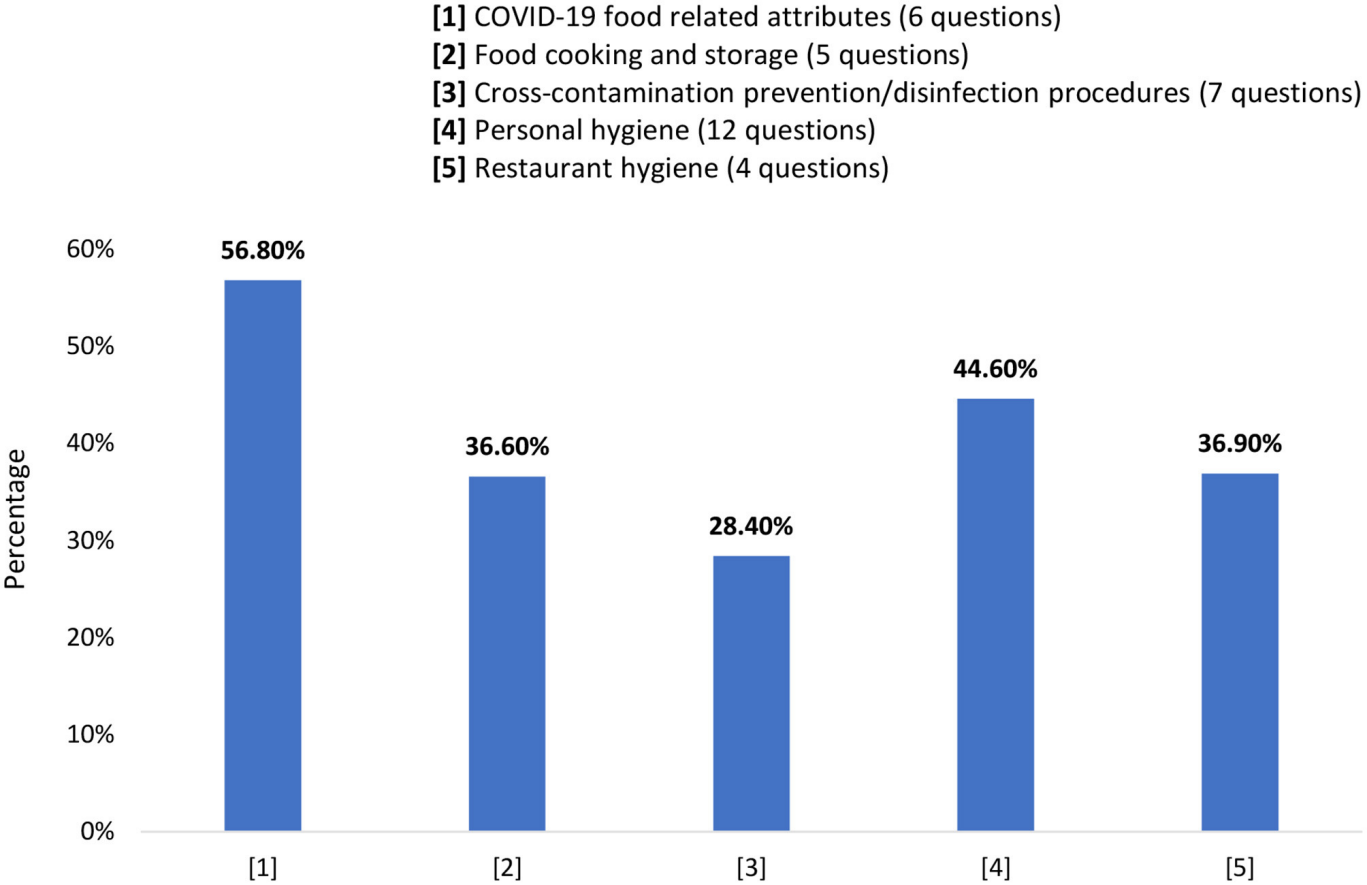
Food safety knowledge, attitudes and practices scores among university students during COVID-19 pandemic (*n* = 1,739).

The overall food safety KAP score of university students during the COVID-19 pandemic was calculated by the summation of correct answers (34 questions) in the tested food safety aspects: “COVID-19 food-related attributes,” “food cooking and storage,” “personal hygiene,” “cross-contamination prevention/disinfection procedures,” and “restaurant hygiene.” The average overall KAP score of the tested aspects was 14.1/34.0 which translates to 41.3% of the questions being answered correctly (Figure 1). The food safety aspect with the highest percentage of correct answers was for “COVID-19 food-related attributes” (56.8%) while the aspect with the lowest percentage of correct answers was “cross-contamination prevention/disinfection procedures” (28.4%).

### COVID-19 Food Related Attributes

Query statements and responses of the COVID-19 food related attributes are presented in Table 2. More than 75% of the respondents possessed the knowledge that the COVID-19 virus flourishes in the nose and mouth of the infected person and it could be transmitted upon coughing or sneezing. A good number of participants (70%) correctly believed that the vaccine solitarily would not be protective against the COVID-19 infection, without compliance to general safety measures (masks, gloves etc.). More than half (62.4%) of the participants correctly believed that COVID-19 does not grow in food; however, only 27.0% believed that it cannot be transmitted through it. A similar number (28.8%) knew that COVID-19 virus could not be found in drinking water (Table 2).

**Table 2:** Query statements and responses of “COVID-19 food-related attributes” aspect.

Question type^*^	**Query statement**	**Frequency (%)**
K	COVID-19 can be found in the nose and mouth of infected person	1325 (76.2%)
K	A person infected with COVID-19 virus without symptoms can transmit the virus when coughing or sneezing to others	1328 (76.4%)
K	COVID-19 virus cannot be found in drinking water	500 (28.8%)
A	I do not think COVID-19 virus can be transmitted through food	470 (27.0%)
A	I do not think COVID-19 virus grows in food	1086 (62.4%)
A	I do not think COVID-19 vaccine will protect me from getting infected when eating outside the home (with friends or in restaurants) without complying to the general safety measures	1218 (70.0%)

* *Knowledge (K), Attitude (A), or Practice (P)*.

### Food Cooking and Storage KAP During the COVID-19 Pandemic

Regarding the appropriate temperature for killing viruses such as COVID-19 during cooking, only 33.2% knew the correct answer (Table 3). And, about 33.5% believed that cooling food in a refrigerator or keeping it in the freezer was ineffective in inhibiting or killing COVID-19. Only 17.0% knew that the best way to check for meat readiness was with the help of a food thermometer. However, the majority of our respondents (89.9%) believed that the number of people involved in food preparation should be reduced in an event where a family member is infected with COVID-19. Moreover, majority (90.4%) of the students did not wash the animal products like eggs before storing them in the refrigerator.

**Table 3:** Query statements and responses of “food cooking and storage” aspect.

Question type^*^	**Query statement**	**Frequency (%)**
K	The appropriate temperature for killing viruses such as COVID-19 virus during cooking is 70 °C	578 (33.2%)
K	The best way to check that poultry is sufficiently cooked is through checking with a thermometer	295 (17.0%)
A	I do not think that cooling food in refrigerator or keeping it in the freezer is effective in inhibiting or killing COVID-19 virus	582 (33.5%)
A	I think that number of people involved in preparing food should be reduced in the event where a family member is infected with COVID-19 virus	1563 (89.9%)
P	During COVID-19 pandemic, I do not wash animal products such as eggs before storing them in the refrigerator	1572 (90.4%)

* *Knowledge (K), Attitude (A), or Practice (P)*.

### Cross-Contamination Prevention/Disinfection Procedures KAP During the COVID-19 Pandemic

In general, the results indicated very low KAP score with respect to cross-contamination prevention and disinfection procedures (28.4%) amongst the students. A very small percentage of the participants (19.8%) were aware about washing of vegetables under running water prior to usage (Table 4). Approximately, 58.1% of the respondents agreed that using the same chopping board to cut vegetables (post raw meat cutting) resulted in cross-contamination. A quarter of the participants (25.4%) falsely believed that using salt, vinegar, pepper or lemon juice was effective in destroying COVID-19 on food-contact surfaces. However, only 42.0% of our respondents knew the correct procedure for cleaning the kitchen surfaces. Less than quarter of our respondents disposed empty shopping bags (19.0%) and disinfected food packages prior to use (23.0%). A lower percentage (11.5%) of our participants used separate sponges for the dishes and the sink.

**Table 4:** Query statements and responses of “cross-contamination prevention/disinfection procedures” aspect.

Question type^*^	**Query statement**	**Frequency (%)**
K	The correct way to wash vegetables is to wash them with running water	345 (19.8%)
K	At home, the proper procedure when cutting vegetables on a cutting board that was previously used for cutting raw meat is to use another board	1010 (58.1%)
K	The proper procedure for cleaning kitchen surfaces is that surfaces are washed with a detergent, rinsed with water, and then wiped with a sterile solution	730 (42.0%)
A	I do not think that using salt, vinegar, pepper or lemon juice is effective in removing germs such as COVID-19 virus from food-contact surfaces	442 (25.4%)
P	During COVID-19 pandemic, I dispose all of shopping bags after emptying their contents	331 (19.0%)
P	During COVID-19 pandemic, I disinfect food packaging or boxes before use	400 (23.0%)
P	During COVID-19 pandemic, I use a separate dishwasher sponges for both dishes and sink	200 (11.5%)

* *Knowledge (K), Attitude (A), or Practice (P)*.

### Personal Hygiene KAP During the COVID-19 Pandemic

In terms of personal hygiene, due to the pandemic, all the respondents recorded not using a mobile phone while preparing food (OR = −0.013, CI = −0.061–0.035), and not using bare hands (OR = −0.133, CI = −0.194-−0.071) while sharing a dish with several people (a common Arab custom), in other words all the respondents used a spoon while sharing a dish with several people (Table 5). As a response to the pandemic, approximately, 90% of our respondents knew that washing hands after handling raw food would aid in reduction of microbial transfer. About half of our respondents (51.8%) agreed that it is was necessary to wash hands after touching the face during food preparation in an effort to prevent spread of the virus. About 44% of our respondents reported washing their hands after touching the outer bags and covers, upon returning home (52.4%), prior to food preparation (43.5%), and eating during the COVID-19 pandemic (51.3%). However, only 36.2% of our participants knew the appropriate duration of handwashing. Approximately, 74 and 78% of our respondents did not agree that hand sanitizers could replace hand washing and knew the best way to dry hands post washing (using a tissue), respectively (Table 5). Only 15.2% of our respondents reported wearing gloves when touching raw food.

**Table 5:** Query statements and responses of “personal hygiene” aspect.

Question type^*^	**Query statement**	**Frequency (%)**
K	While preparing food, hands should be washed after touching the face	900 (51.8%)
K	Washing hands after handling raw food reduce the transmission of food-related germs	1562 (89.8%)
K	20 seconds is sufficient to wash hands	630 (36.2%)
K	The best way to dry my hands after washing them is to use a tissue paper	1347 (77.5%)
A	I do not think that using hand sanitizers should replace washing hands with soap and water to get rid of germs	1278 (73.5%)
P	During COVID-19 pandemic, I wash my hands before eating	892 (51.3%)
P	During COVID-19 pandemic, I do not use hands to eat directly without a spoon while sharing the dish with several people	1739 (100%)
P	During COVID-19 pandemic, I wash my hands after touching the outer bags and covers	765 (44.0%)
P	During COVID-19 pandemic, I wash my hands when I get home	911 (52.4%)
P	During COVID-19 pandemic, I wash my hands before preparing food	756 (43.5%)
P	During COVID-19 pandemic, I do not use my mobile phone while preparing food	1739 (100%)
P	During COVID-19 pandemic, I wear gloves when touching raw (uncooked) food	265 (15.2%)

* *Knowledge (K), Attitude (A), or Practice (P)*.

### Restaurant Hygiene Behavior in Response to COVID-19

Regarding restaurant hygiene during the COVID-19 pandemic, 35.0% of the university students checked tables and chairs (if they were sanitized) before sitting, 38.9% checked the bathroom (for sanitization) before using it, 39.6% paid attention to the safety measures taken by workers at restaurants, such as the use of masks, gloves and physical distancing, and 34.2% observed whether the restaurant followed social distancing protocols for visitors (Table 6).

**Table 6:** Query statements and responses of “restaurant hygiene” aspect.

Question type^*^	**Query statement**	**Frequency (%)**
P	When going to a restaurant, I check the disinfection procedures for tables and chairs before sitting during COVID-19 pandemic	608 (35.0%)
P	When going to a restaurant, I check the disinfection procedures in the bathroom before using it during COVID-19 pandemic	676 (38.9%)
P	When going to a restaurant, I check the sanitation and safety measures of workers, such as masks, gloves and physical distancing during COVID-19 pandemic	689 (39.6%)
P	When going to a restaurant, I make sure that the restaurant applies the condition of social distancing between visitors during COVID-19 pandemic	595 (34.2%)

* *Knowledge (K), Attitude (A), or Practice (P)*.

### Behavioral Changes During the COVID-19 Pandemic

**Figure 2:**
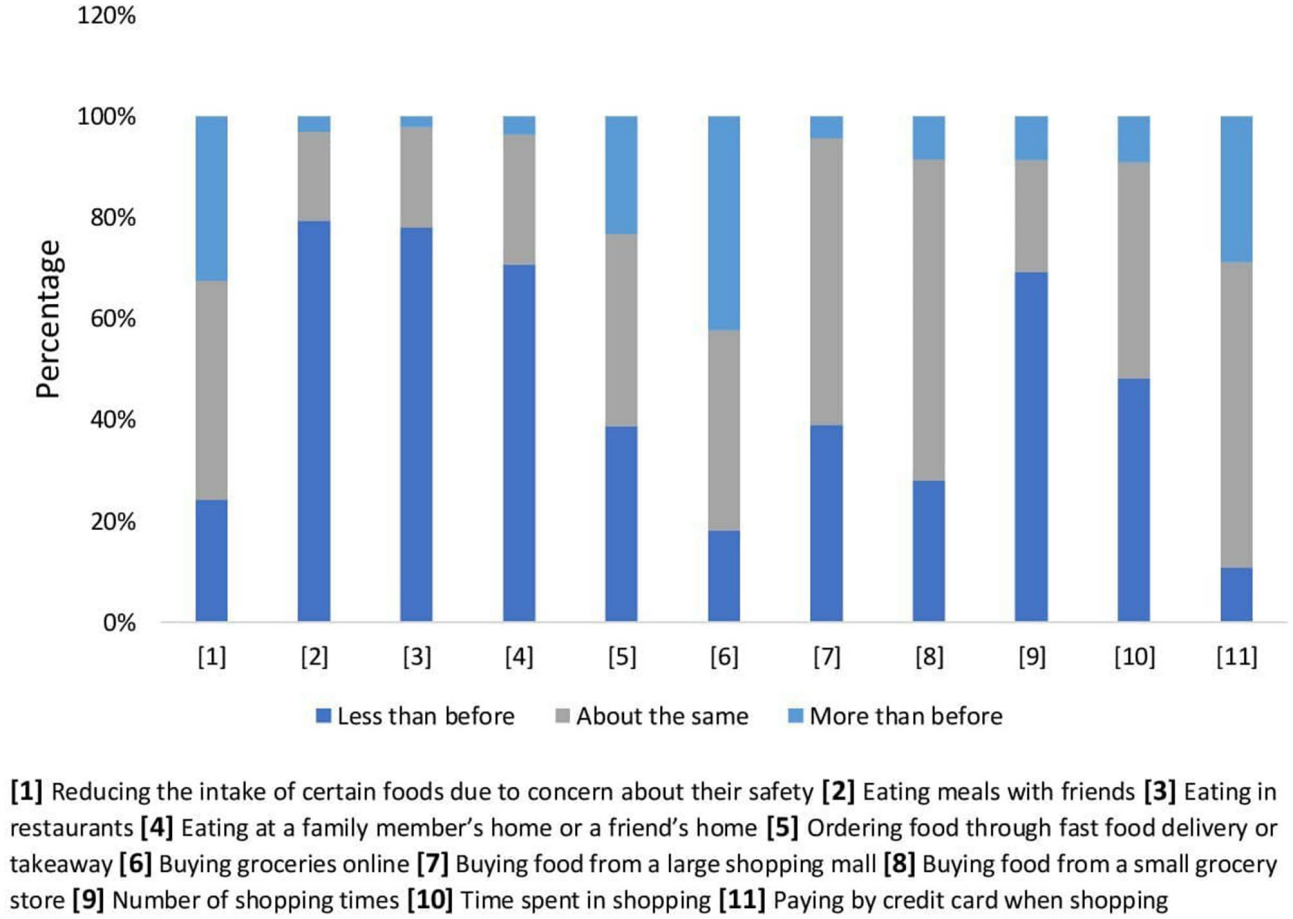
Changes of food-related behaviors during COVID-19 pandemic (*n* = 1,739).

The results regarding food-related practices during COVID-19 pandemic suggest clear changes in student behaviors. As shown in Figure 2, 79.5 and 70.8% of the participants reported reduced eating and gathering with friends and family members, respectively during the COVID-19 pandemic. Moreover, 78.0% of respondents reported dining out less than before. In this study about half (42.4%) of the students shifted toward buying groceries online and only 28.9% of participants paid their bills by credit card more than that before the pandemic. In our study, buying from a large shopping mall or a small grocery store stayed approximately the same while comparing the pre and post pandemic periods. However, 69.2% of our participants reduced the frequency of their shopping visits while another 48.1% reduced the time spent during shopping because of the pandemic (Figure 2).

### The Association Between Overall Food Safety Knowledge, Attitudes, and Practices Scores and General Characteristics of University Students During the COVID-19 Pandemic

In this study, no significant (*P* ≥ 0.05) association was observed between overall food safety knowledge, attitudes, and practices **(**KAP) score of university students during the COVID-19 pandemic and age, university type, college, studying year, living status and the enrollment in a food preparation course (Table 7). Significant associations (*P* < 0.05) were found between respondents' food safety KAP scores and gender, marital status, university degree, employment status, self-rating of food safety knowledge, and the source of food safety information. The current study found that females (18.0%) had higher food safety KAP scores than males (5.6%). In other words, 18.0% of all female participants answered more than half of the questions correctly. In the current study, being a female was not only significantly associated with higher food safety KAP scores but also was a predictor that effected KAP results. Married students in this study scored a higher KAP (*P* < 0.05) than single students; more than one third of the married participants answered more than 50% of the questions correctly while less than a quarter of the single participants got correct answers. The current study showed that there was a direct relationship (*P* < 0.05) between the educational level and KAP scores. Higher education program (Masters) students had a higher score than their undergraduate counterparts (2.6 vs. 21% respectively). Students who work in part time jobs had higher (*P* < 0.05) KAP scores compared to full time and unemployed students. This study showed a strong association (*P* < 0.05) between self-rated food safety knowledge and KAP scores. Those who rated themselves to have higher knowledge indeed got higher KAP scores. Majority of the students in this study agreed that their major source of food safety information was the Internet (43.2%) followed by family (22.3%). In this study taking courses/workshops and consulting a healthcare professional about food safety information were significantly associated with higher (*P* < 0.05) food safety KAP scores.

**Table 7:** Association between food safety knowledge, attitudes, and practices scores and general characteristics of university students.

**Character**	Good KAP^*^	**Poor KAP**	*P*-value^**^
Age	411 (23.6%)	1328 (76.4%)	*P* = 0.168
Mean ± SD	21.39 ± 1.80	21.22 ± 1.76	
**Gender**			
Male	98 (5.6%)	473 (27.2%)	*P* <0.001
Female	313 (18.0%)	855 (49.2%)	
**Marital status**			
Single	372 (21.4%)	1258 (72.3%)	*P* = 0.002
Married	39 (2.2%)	70 (4.0%)	
**University type**			
Public	235 (13.5%)	771 (44.3%)	*P* = 0.752
Private	176 (10.1%)	557 (32.0%)	
**College**			
Humanities	125 (7.2%)	434 (25.0%)	*P* = 0.117
Scientific	150 (8.6%)	525 (30.2%)	
Health	136 (7.8%)	369 (21.2%)	
**University degree**			
Bachelors	365 (21.0%)	1239 (71.2%)	*P* = 0.003
Masters	46 (2.6%)	85 (4.9%)	
Doctorate	0 (0.0%)	4 (0.2%)	
**Year of study**			
First	88 (5.1%)	251 (14.4%)	*P* = 0.625
Second	66 (3.8%)	231 (13.3%)	
Third	95 (5.5%)	297 (17.1%)	
Fourth	114 (6.6%)	399 (22.9%)	
Fifth	37 (2.1%)	102 (5.9%)	
Sixth	11 (0.6%)	48 (2.8%)	
**Living with**			
Family	372 (21.4%)	1178 (67.7%)	*P* = 0.404
Roommate	14 (0.8%)	66 (3.8%)	
Alone	25 (1.4%)	84 (4.8%)	
**Employment**			
Do not work	299 (17.2%)	1047 (60.2%)	*P* = 0.004
Full time work	43 (2.5%)	139 (7.9%)	
Part time work	69 (4.0%)	142 (8.2%)	
Monthly expenses
<100 JD	190 (10.9%)	655 (37.7%)	*P* = 0.533
100–300 JD	175 (10.1%)	528 (30.4%)	
>300 JD	46 (2.6%)	145 (8.3%)	
**Self-rating of food safety knowledge**			
Excellent	64 (3.7%)	144 (8.3%)	*P* <0.001
Very good	181 (10.4%)	444 (25.5%)	
Good	152 (8.7%)	616 (35.4%)	
Weak	12 (0.7%)	110 (6.3%)	
Very weak	2 (0.1%)	14 (0.8%)	
**Source of food safety information**			
Courses/workshops	103 (5.9%)	186 (10.7%)	*P* <0.001
Family	73 (4.2%)	315 (18.1%)	
Friends	1 (0.1%)	25 (1.4%)	
Healthcare professional	28 (1.6%)	57 (3.3%)	
Social media	35 (2.0%)	132 (7.6%)	
Internet	165 (9.5%)	587 (33.8%)	
Others^*^	6 (0.3%)	26 (1.5%)	
**Preparing/helping in preparing food**			
Yes	355 (20.4%)	1098 (63.1%)	*P* = 0.077
No	56 (3.2%)	230 (13.2%)	

* *Good KAP score: >50% of the questions were answered correctly.*

** *Significance level at P < 0.05*.

Logistic regression results (Table 8) showed that male respondents had a lower Odds ratio compared to females (0.5) (*P*-value < 0.05). This analysis indicated that males were 0.5 times less likely to have good KAP scores than females. Moreover, in this study, unemployed and full-time employee students were 0.6 times less likely to have good KAP scores in comparison with part time employee students. This finding was unique to our study and has not been observed in previously published work to the best of our knowledge.

**Table 8:** Predictors of food safety KAP using logistic regression analysis.

**Character**	**OR (CI)**	*P*-value^*^
**Gender**		
Male	0.464 (0.343–0.629)	*P* <0.001
Female	Reference	
**Marital status**		
Single	0.815 (0.332–2.002)	*P* = 0.655
Married	Reference	
**Employment**		
Do not work	0.637 (0.407–0.995)	*P* = 0.048
Full time work	0.588 (0.429–0.805)	*P* = 0.001
Part time work	Reference	
**Self-rating of food safety knowledge**		
Excellent	Reference	
Very good	2.303 (0.498–10.663)	*P* = 0.286
Good	2.451 (0.544–11.041)	*P* = 0.243
Weak	1.46 (0.324–6.569)	*P* = 0.622
Very weak	0.786 (0.156–3.968)	*P* = 0.77
**Source of food safety information**		
Courses/workshops	Reference	
Family	2.202 (0.799–6.066)	*P* = 0.127
Friends	1.226 (0.445–3.377)	*P* = 0.693
Healthcare professional	0.278 (0.029–2.628)	*P* = 0.264
Social media	2.16 (0.722–6.466)	*P* = 0.169
Internet	1.5 (0.519–4.331)	*P* = 0.454
Others	1.451 (0.536–3.923)	*P* = 0.464

* *Significance level at P < 0.05*.

## Discussion

This study aimed to investigate the level of KAPs of Jordan universities students during COVID-19 pandemic. Gaps in food safety knowledge, attitudes and practices were identified in this population, as the participants were found to have insufficient scores of overall food safety KAP. This level of food safety knowledge amongst university students has been previously reported (14, 16, 22, 23, 30). A meta-analysis reported overall KAP scores regarding COVID-19 to be 78.9, 79.8, and 74.1, respectively (31).

COVID-19 transmission route is reported to be either by person to person contact or via droplet transfer upon sneezing and coughing (12), the majority of our participants knew the way of COVID-19 transmission. In Saudi Arabia, it was reported that 94.8% of the participants knew that COVID-19 spread could be via the transfer of respiratory droplets upon coughing or sneezing, and only a small number (14.9%) knew that infected people with no fever could transmit the virus to others (32). While, amongst the South East Asian consumers, about half of the respondents (50.3%) were unaware that asymptomatic infected food handlers could transmit COVID-19 (33). The respondents of this study wrongly knew that food and water were vehicles for virus transfer. Official records report that there is no evidence that people can be infected with COVID-19 via food or water consumption, as it is a respiratory disease. Moreover, COVID-19 cannot multiply in foods (as correctly thought by our respondents), as the viruses need a human or an animal host to grow (34). A large number of participants believed that the vaccine alone will not be protective against getting infected without complying to safety measures, CDC and WHO also recommend following safety precautions at public places even after being fully vaccinated (35, 36).

With regards to food cooking and storage during the COVID-19 pandemic, KAP score about appropriate cooking temperature for killing viruses was relatively low. South East Asian population showed that (41.2%) of participants believed that cooking at a temperature of >70°C destroyed the coronavirus (33). It is documented that coronavirus is a thermolabile virus and it is susceptible to traditional cooking temperatures (70°C) (37). However, when talking about refrigeration and freezing one third of our participants believed that these cooling techniques is not effective against COVID-19 virus. This proportion is lower than previously published studies where 64 and 52% of university students in Lebanon and Jordan knew that freezing does not kill harmful germs in food, respectively (16, 22). The authors expected respondents to cook meat well as a response to the belief that the heat would kill the virus present in the meat. Hence, it was expected that they use a thermometer to check for meat wellness. Contrary to the assumption, students demonstrated a low knowledge about using a food thermometer as an accurate way of determining whether meat are cooked enough to prevent food poisoning. Previous studies also testify to this premise; university students were reported to have limited knowledge about the suggested use of food thermometer for such a purpose (16, 22, 23, 38). Our results showed a good attitude toward food preparing situations which harmonizes with the general recommendation from WHO to limit the number of persons involved in food preparation during COVID-19 pandemic (12). Mishandling of food can occur at any stage during preparing and storage, for example, not washing the eggs is encouraged as washing could make them more porous and would result in microbial transfer to the internal section of the egg (39). The authors expected the respondents to wash animal products like eggs prior to storage as a precautionary measure to combat the virus, but surprisingly the majority of students did not.

Surfaces contaminated with COVID-19 may act as vehicles for spread of the virus. The virus could be present on chopping boards, knives etc. More than half of the students knew that they should use different chopping boards for vegetables and meat. This is in accordance with previous studies (23, 38, 40). Students displayed poor knowledge regarding cross contamination prevention and disinfection procedures. For instance, the respondents have a poor knowledge about the correct way to wash vegetables, where running water is expected to aid in washing away the virus. The majority of respondents have wrong information about the use of salt, vinegar, pepper and lemon juices as cleaning items. Such measures have officially been reported to be ineffective (34). Cleaning surfaces with detergent, water and then a disinfectant would be the most appropriate way for reducing the presence of the virus on kitchen surface tops. The authors expected students to dispose/disinfect shopping bags/ other food packaging to prevent virus transfer from outside to homes. Most of the respondents did not dispose empty shopping bags and disinfect food packaging, a similar pattern was observed in Jordanian participants where only 15.2% of the reported disposing of all boxes, packages, and covers of food while 13.4% reported always disinfecting food packaging prior to home storage (10). In contrast, about 40% of the consumers in Indonesia and Malaysia washed or wiped food jars and cans before using them (33), and 71.9% of United Arab Emirates residents sanitized or cleaned groceries before storing them (41). A higher percentage of our participants have insufficient knowledge on the proper use of reusable kitchen sponges as they reported using them for multiple purposes such as dishes and sink. This finding contrasts with a previous study that showed that a high percentage (74%) of female students in university dormitories used different sponges for cleaning utensils and the sink (26).

Unexpectedly, our university students exceeded other populations in not using their mobile phones while preparing food, and not using hand in a one-dish shared meal. Our results differ considerably from another study which reported 81.4% of the respondents used their cellphone during food preparation, cooking and packaging (42). It is expected that consumers sans a pandemic would use their cell phones during food preparation for various purposes (checking recipes, posting food pictures etc.); however, as the cellphone/ spoon could have remnant virus on its surface, if not disinfected, the respondents seem to exercise caution, which is encouraging. The majority of the respondents agreed that washing hands regularly and after touching the face and raw foods is important in preventing COVID-19 spreading. Similarly, Italian undergraduate students agreed that handwashing, wearing masks and avoiding close contacts were good protective measures to prevent the spread of COVID-19 (43). Only half of the respondents wash their hands before eating, and the same percentage wash their hands after returning home. It is not only handwashing but rather the time spent doing this activity that is equally important. In North Central Nigeria, majority (82.3 %) of respondents agreed that handwashing should last from a minimum of 20 s to 1 minute (44). Less than half of our participants knew how long they should wash their hands. This is a matter of grave concern as handwashing is one of the best front-line approaches to combat the virus. The respondents need to be educated about the correct handwashing technique/ time. In a multi-country study, 36.3% of Jordanian community washed their hands after returning home before the COVID-19 pandemic, this percentage increased to 53% during COVID-19 (10). This is still a low number considering the perilous behavior of this virus. A large number of participants have a good knowledge about the effectiveness of hand sanitizers but agreed with the need for hand washing. This is in accordance with the WHO recommendations which highlight that hand sanitizers should not replace washing hands with water and soap (12). It is possible that the virus transfers from the contaminated food surface to the respondent's hand which could then infect the person via oral orifices. The respondents were hence expected to use gloves while handling raw foods. It was noted that only a small number (*n* = 265) wore gloves when dealing with raw foods. On similar lines, 98.4% Philippine food handlers who were engaged in an online food business, reported that they did not use gloves when handling raw food during the COVID-19 pandemic (42). However, it is agreed upon that although gloves are an important hygienic measure, they cannot replace hand washing. Hands need to be washed prior to wearing gloves and also after their removal (45).

Approximately more than one third of the participants checked restaurants hygienic measures such as tables, chairs and toilet sanitization, as well as workers' safety precautions. CDC recommends the use of masks for both employees and customers (46). It is obvious that costumers would be more confident about going to restaurants, if the restaurant management followed hygienic/sanitizing practices besides mandating workers to wear masks and maintain social distancing (25). More than half (57.4%) of the consumers in Indonesia and Malaysia always choose to dine in restaurants that followed social distancing rules, and 37.6% always sanitized the utensils and tables before eating at restaurants (33). Most (93%) of the customers in the study expected some safety precautions by restaurants, such as hand sanitizers at the door, staff adherence to masks and gloves, social distancing and reduced costumer serving capacity (47). Such measures along with toilet disinfection, surface sanitization and ventilation limit the spread of the COVID-19 virus (48).

Indeed, there have been a noted change in students' behavior toward gatherings, eating with family and friends during COVID-19. In Qatar, people reported eating more with immediate family members during COVID-19 (20). The author highlighted a shift toward eating meals at home rather than restaurants and a significant increase in home food deliveries during the COVID-19 pandemic. A similar trend was seen in Netherlands too during the pandemic lockdown, with 29.5% of the participants using meal delivery services more frequently than usual (49). It was reported that young, educated adults, tended to use internet services like online grocery shopping and meal delivery more frequently compared to their older counterparts (20). A shift toward using an online grocery delivery is shown in the results, however, more than half of the participants did not use credit cards as a safe payment method. Payment by credit card was expected to be preferred as cash could act as a vehicle for virus exchange.

Students reported shopping from either small grocery stores or large supermarkets as before but they reduced their time and frequency of shopping. However, in an Italian community, a shift toward shopping from small grocery stores due to the pandemic was observed. This may be because small grocery stores are less crowded than large supermarkets and hence are preferred by consumers (50). During the pandemic, Spanish consumers showed a significant reduction in the frequency of shopping; however, no significant change in the food shopping location was recorded (51).

Regarding the relationship between the KAP scores and demographic characteristic, this study shows that gender, marital status, university degree, employment status, self-rating of food safety knowledge, and the source of food safety information have a significant association. Female respondents outnumbered their male counterparts in KAP scores, this result might be related to the fact that traditionally in Jordan females play a central role in food preparation, kitchen work, cleaning, as well as the cultural trend of mothers passing their food related experience to daughters. A study of university students in Indiana showed that females had a higher food safety knowledge mean score (7.41) than males (7.04) (52). However, a Greek study showed that both genders had the same knowledge level about food safety issues (23). While, female and male Lebanese university students showed an equal knowledge level about food safety; however, female students had better food safety practices (22). Students identified as married in this study obtained higher KAP scores than single students. This is probably because married couples need to take charge of housekeeping and food preparation. In contrast, in Kuwait, single students were observed to get higher scores in food handling practices compared to their married counterparts. This could probably because in the country, married couples traditionally live in an extended family home and they tend to hire domestic helpers who aid in food preparation (53).

Higher education programs students reported higher KAP scores, this can be attributed to the greater amount of knowledge of these students by their readings, studying and experience. Part-time jobs have been considered as one of the factors influencing students KAP scores. Their work experience may have contributed to this observation. This factor was also another predictor of food safety KAP results in the present study.

Higher self-rated food safety knowledge levels correspond with higher KAP scores. A similar observation amongst college and university students in the United States was observed that the lower the self-rated food safety knowledge level, the lower was the knowledge mean scores (54). Common major sources of food safety knowledge among participants are internet and family, these were also the main sources of information in a study (40). However, another study reported that people tended to trust healthcare professionals more about COVID-19 related information (55). Food safety information from courses/ workshops and healthcare professionals also correspond with higher KAP scores. Swedish university students who reported food safety education as their primary source of knowledge answered a higher number of food safety knowledge questions correctly (40). On the other hand, being informed by family about food safety was related to poorer food preparation safety knowledge (23, 38).

The present study was limited to students who have access to social media since it was conducted online.

## Conclusion

University students in Jordan have insufficient scores in terms of overall food safety knowledge, attitudes and practices, a matter of great concern especially during the COVID-19 pandemic. However, results of this study report positive behavioral changes due to the pandemic with study participants increasing the adoption of hygienic practices. Fundamentals of food safety should be implemented in university curricula to better educate young adults.

## Study Strength and Limitations

A current very important topic related to COVID-19 and food safety has been addressed in this manuscript. As the sample size was high, the generalizability of the results was at a good level. However, the findings of the study confer to the Jordanian students alone. Perhaps students from other countries would rate differently. Moreover, the survey questions pertaining to “practices” are subject to recall. Errors in recollection in terms or practice may have resulted in bias.

## Data Availability Statement

The raw data supporting the conclusions of this article will be made available by the authors, without undue reservation.

## Ethics Statement

The studies involving human participants were reviewed and the protocol were approved by the Department of Nutrition and Food Technology (#26/2021) and Deanship of Graduate Studies (#7/2021) at Jordan University of Science and Technology. The patients/participants provided their written informed consent to participate in this study.

## Author Contributions

TO, AA-N, and AT contributed to conception and design of the study. TO and AT contributed to manuscript writing. All authors contributed to manuscript revision, read, and approved the submitted version.

## Conflict of Interest

The authors declare that the research was conducted in the absence of any commercial or financial relationships that could be construed as a potential conflict of interest.

## Publisher's Note

All claims expressed in this article are solely those of the authors and do not necessarily represent those of their affiliated organizations, or those of the publisher, the editors and the reviewers. Any product that may be evaluated in this article, or claim that may be made by its manufacturer, is not guaranteed or endorsed by the publisher.
