# Supplementary material for: Food Safety Knowledge, Attitudes, and Practices Among Jordan Universities Students During the COVID-19 Pandemic
Source: Front Public Health. 2021 Aug 30;9:729816. doi: 10.3389/fpubh.2021.729816 (PMC8435670; doi:10.3389/fpubh.2021.729816)
Supplement: Supplementary file 1 [file Table_1.DOCX]

**Food safety knowledge, attitudes and practices among Jordan universities students during the Covid-19 pandemic**

**Demographic data**

Age:

- 18
- 19
- 20
- 21
- 22
- 23
- 24
- 25+

Gender:

- Female
- Male

Marital status:

- Single
- Married

University:

- Jordan University of Science and Technology
- The University of Jordan
- Yarmouk University
- Mutah University
- The Hashemite University
- Al Albayt University
- AlBalqa Applied University
- Al-Hussein Bin Talal University
- Tafila Technical University
- German Jordanian University
- Princess Sumaya University for Technology
- Isra University
- American University of Madaba
- University of Petra
- Amman Arab University
- AlZaytoonah University of Jordan
- Middle East University
- Jadara University
- Al-Ahliyya Amman University
- Applied Science University
- Philadelphia University
- Zarqa University
- Irbid National University
- Jerash Universtiy
- Ajloun National University
- Aqaba University of Technology
- AlHussein Technical University
- The World Islamic Sciences and Education University
- Arab Open University

College:

- Humanities
- Scientific
- Health

University degree:

- Bachelor
- Master
- PhD

Year of study:

- First year
- Second year
- Third year
- Fourth year
- Fifth year
- Sixth year

Living with:

- Family
- Roommate
- Alone

Employment:

- Do not work
- Full-time work
- Part time work

Monthly expenses:

- <100 JD (1 JD = 1.41$)
- 100-300 JD
- >300 JD

Self-rating of food safety Knowledge:

- Excellent
- Very good
- Good
- Weak
- Very weak

Source of food safety information:

- Courses/workshops
- Family
- Friends
- Health care professional
- Social media
- Internet
- Others

Preparing/helping in preparing food

- Yes
- No

**Food safety knowledge during Covid-19**

Can COVID-19 be found in the nose and mouth of infected person?

- Yes
- No
- Not sure

Can a person infected with COVID-19 virus without symptoms transmit the virus when coughing or sneezing to others?

- Yes
- No
- Not sure

Can COVID-19 virus be found in drinking water?

- Yes
- No
- Not sure

What is the appropriate temperature for killing viruses such as COVID-19 virus during cooking?

- 30ºC
- 50 ºC
- 70 ºC
- Not sure

What is the best way to check that poultry is sufficiently cooked?

- When the juice runs clear
- Taste
- Look
- When it has the correct food thermometer reading
- Not sure

Does washing hands after handling raw food reduce the transmission of food-related germs?

- Yes
- No
- Not sure

For how long is it sufficient to wash hands?

- 10 seconds
- 20 seconds
- 30 seconds
- 40 seconds
- Not sure

What is the best way to dry your hands after washing them?

- Tissue paper
- Hot air electrical dryer
- You leave them to dry on their own
- Your clothes
- Not sure

While preparing food, hands should be washed after touching?

- Your face
- Clean pots and counter
- Utensils being used in food preparation
- It is not necessary wash my hands after touching any of the above
- Not sure

What is the correct way to wash vegetables?

- Water and salt
- Regular soap
- Hot water
- Cold running water
- Not sure

At home, what is the proper procedure when cutting vegetables on a cutting board that was previously used for cutting raw meat?

- Use the cutting board as it is
- Wipe the cutting board off with a paper towel
- Rinse the cutting board under water and soap
- Use another cutting board
- Not sure

What is the proper procedure for cleaning kitchen surfaces?

- Spray with a strong sanitizing solution
- Wash with a detergent, rinse, then wipe with a sanitizing solution
- Wipe with a sanitizing solution, then rinse with clean water and wipe dry
- Brush off any dirt or food pieces, then wipe with sanitizing solution
- Not sure

**Food safety attitudes during Covid-19**

Do you think COVID-19 virus can be transmitted through food?

- Yes
- No
- Not sure

Do you think COVID-19 virus grows in food?

- Yes
- No
- Not sure

Do you think that cooling food in the refrigerator or keeping it in the freezer is effective in inhibiting or killing Covid-19?

- Yes
- No
- Not sure

Should the number of people involved in preparing food be reduced in the event where a family member or a friend is infected with Covid-19?

- Yes
- No
- Not sure

Do you think that using salt, vinegar, pepper or lemon juice is effective in removing germs such as Covid-19 from surfaces which are in direct contact with food?

- Yes
- No
- Not sure

Do you think that using hand sanitizers should replace washing hands with soap and water to get rid of germs?

- Yes
- No
- Not sure

Do you think COVID-19 vaccine will protect you from getting infected when eating outside the home (with friends or in restaurants) without complying to the general safety measures?

- Yes
- No
- Not sure

**Food safety practices during Covid-19**

During Covid-19, do you eat meals with friends?

- Less than before
- About the same
- More than before

During Covid-19, do you eat in restaurants?

- Less than before
- About the same
- More than before

During Covid-19, do you eat at a family member’s home or a friend’s home?

- Less than before
- About the same
- More than before

During Covid-19, do you order food through fast food delivery or takeaway?

- Less than before
- About the same
- More than before

During Covid-19, do you wash your hands before eating?

- Yes
- No

During Covid-19, do you use your hands to eat directly without a spoon while sharing the dish with several people?

- Yes
- No

During Covid-19, do you reduce the intake of certain foods due to your concern about their safety?

- Less than before
- About the same
- More than before

During Covid-19, do you buy groceries online?

- Less than before
- About the same
- More than before

During Covid-19, do you personally go to buy food from a large shopping mall (Safeway, Carrefour, ...)?

- Less than before
- About the same
- More than before

During Covid-19, do you personally go to buy food from a small grocery store?

- Less than before
- About the same
- More than before

During Covid-19, did the number of times you go shopping differ?

- Less than before
- About the same
- More than before

How long does it take you to do shopping during Covid-19?

- Less than before
- About the same
- More than before

During Covid-19, do you pay by credit card when shopping?

- Less than before
- About the same
- More than before

During Covid-19, do you wash your hands after touching the outer bags and covers?

- Yes
- No

During Covid-19, do you wash your hands when you get home?

- Yes
- No

During Covid-19, do you wash your hands before preparing food?

- Yes
- No

During Covid-19, do you wear gloves when touching raw (uncooked) food?

- Yes
- No

When going to a restaurant, do you check the sterilization procedures for tables and chairs before sitting during Covid-19 pandemic?

- Yes
- No

When going to a restaurant, do you check the sterilization procedures in the bathroom before using it during Covid-19 pandemic?

- Yes
- No

When going to a restaurant, do you check the sterilization and safety measures for workers, such as masks, gloves and physical distancing during Covid-19 pandemic?

- Yes
- No

When going to a restaurant, do you make sure that the restaurant applies the condition of social distancing between visitors during Covid-19 pandemic?

- Yes
- No

During Covid-19, do you dispose of all shopping bags after emptying their contents?

- Yes
- No

During Covid-19, do you disinfect food packaging or boxes before use?

- Yes
- No

During Covid-19, do you use a separate dishwasher sponge for both dishes and sink?

- Yes
- No

During Covid-19, do you wash animal products such as eggs before storing them in the refrigerator?

- Yes
- No

During Covid-19, do you use your mobile phone while preparing food?

- Yes
- No
